# Spatial distribution of soil organic carbon in an Irish salt marsh (Rogerstown Estuary)

**DOI:** 10.1007/s10661-025-14250-9

**Published:** 2025-07-10

**Authors:** Juliet Rounce, Iris Möller, Andrew J. Manning

**Affiliations:** 1 Department of Geography, Trinity College Dublin, Dublin, Ireland; 2 Coasts and Oceans Group, HR Wallingford, Howbery Park, Wallingford, Oxon OX10 8BA UK; 3 School of Biological and Marine Sciences, University of Plymouth, Drake Circus, Plymouth, Devon PL4 8AA UK

**Keywords:** Coastal, Wetlands, Blue carbon, Saltmarsh, Intertidal

## Abstract

Salt marshes are globally widespread, found on low-lying coastal shores, and are highly effective at long-term carbon storage; thus, they are vital for climate change impact mitigation. Accurate carbon stock estimation requires an understanding of local-scale spatial variability of carbon storage and the facilitating processes. Few studies investigate the cumulative impact of controlling factors on within-site carbon distribution. This study utilises 60 cores from a salt marsh in Turvey Nature Reserve (Rogerstown Estuary), on the Irish east coast, to investigate spatial variability in soil organic carbon (SOC) content, alongside bio-sedimentary, and environmental factors. Mean carbon density (CD) was 11.1 ± 4.2 kg m^−3^ at 10-cm depth, ranging from 5.2 to 22 kg m^−3^ (423% increase) across the marsh. We recommend that to obtain measurements across the full range of the site, for small sample sizes (*n* < 20), random sampling should be used (mean difference between the site-wide CD and ‘subsample CD’ ranged from 0.04 (*n* = 10) to 0.29 (*n* = 5) kg m^−3^) and marsh edge clustering should be avoided. These results provide the first ever systematic record of local-scale (within ~ 800 m^2^) SOC and CD variability within an Irish east coast salt marsh and the variation of known influencing factors (including sedimentary and environmental). We also present the first study to systematically provide guidance on capturing marsh-wide SOC and CD most effectively based on limited sampling. The outputs help constrain uncertainties around scaled-up carbon accumulation estimates for regional, national and international inventories.

**Supplementary Information:**

The online version contains supplementary material available at 10.1007/s10661-025-14250-9.

## Introduction

**Fig. 1:**
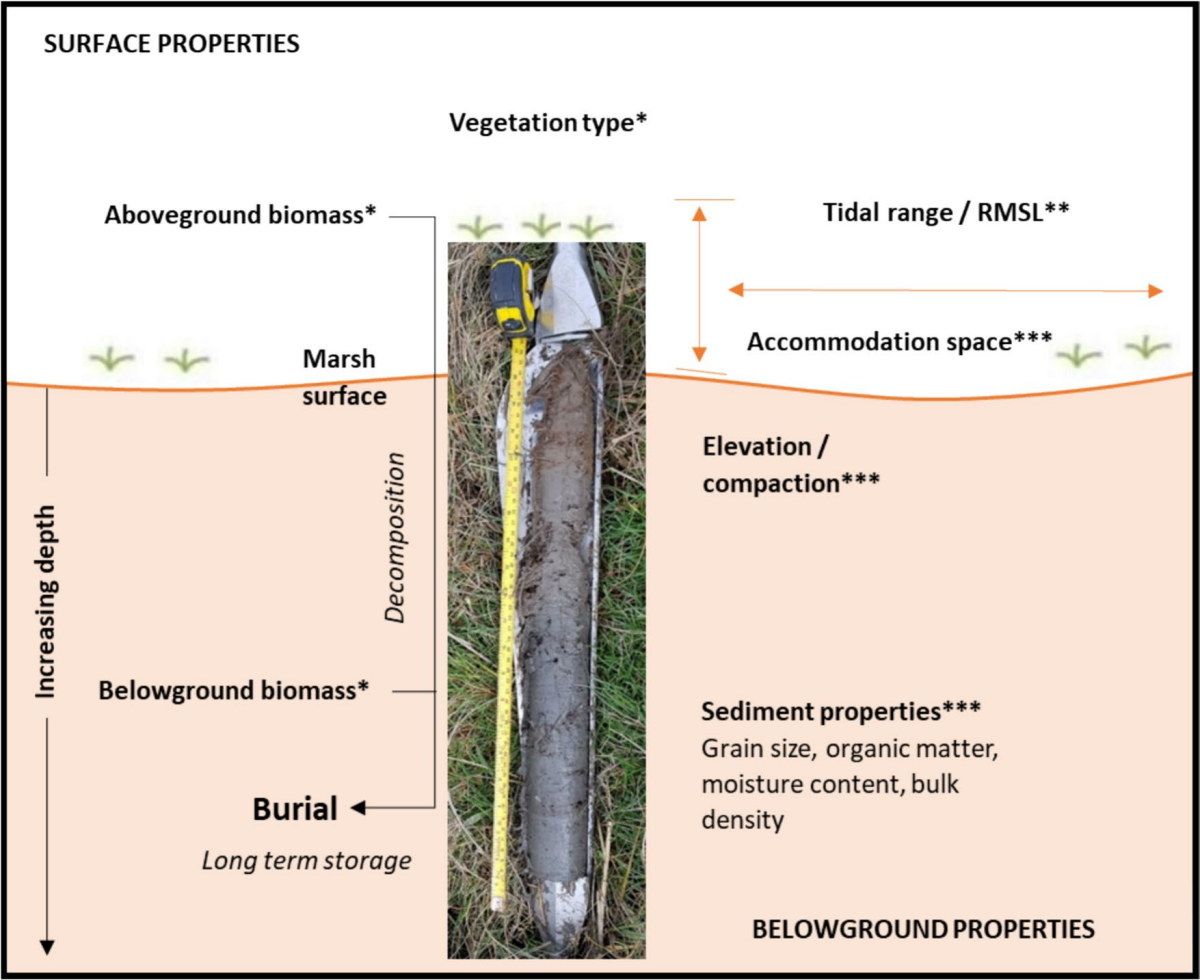
Above- and belowground influencing properties and key measurements in the vertical scale on belowground organic carbon accumulation on an example core from a minerogenic Irish salt marsh. Arrows indicate the space in which properties impact carbon content. Biological (*), hydrodynamic (**) and sedimentary factors (***) are indicated. Burial = key long-term carbon storage process. RMSL = relative mean sea level

Salt marshes are found on every continent except Antarctica, on low-lying coastal shores that form the transitional zone between marine and terrestrial ecosystems (e.g. Perillo et al., 2019). Salt marsh formation and functioning is dependent on interacting biophysical processes (ecological, hydrodynamic and sedimentary) within the tidal frame, forming a complex three-dimensional system (Cahoon et al., 2006; Rounce et al., 2025). Furthermore, salt marshes may exhibit carbon sequestration rates up to twice those occurring in terrestrial forests (Byun et al., 2019; McLeod et al., 2011). By sequestering atmospheric carbon dioxide, salt marshes provide vital climate regulation services (Heckbert et al., 2011). Thus, salt marshes offer a particularly good potential as a long-term carbon sink (centuries timescale), which is dependent on high productivity, anaerobic water-logged soils, reducing the decomposition rate of organic matter (OM), and rapid burial through tidally derived sediments, which demonstrate high stability (Howard et al., 2014; Rounce et al., 2025). As such, in the context of recent national and international carbon emissions targets, the need for accurate determination of the true spatial and temporal variation in belowground stored organic carbon (OC) for OC inventories is rising (e.g. Smeaton et al., 2022a, 2023). Various interdependent factors play a key role in the burial of OC and its distribution (Fig. 1). For example, biological processes, such as vegetation type and distribution (e.g. Howard et al., 2014; Penk & Perrin, 2022); hydrodynamics, relating to conditions which facilitate deposition and/or retention of deposited matter (Moeller et al., 1999, 2001; Schuerch et al., 2019) and sedimentary characteristics and geomorphology (Brooks et al., 2021; Cahoon et al., 2000, 2006; Kelleway et al., 2016; Watts et al., 2003). Distance from marsh margin and to a natural tidal creek can be proxies for environmental factors also play a vital role in the control of OC distribution (e.g. Allen, 2000; Kim et al., 2013; Miller et al., 2023; Reed, 1999; Roner et al., 2016; Russell et al., 2024).

### Carbon stock estimation and variation

Carbon stocks (as an amount per area) refer to the measurement of OC in the ecosystem to a defined depth (see Howard et al., 2014). Carbon is stored in aboveground (AGB) and belowground plant biomass (BGB), as well as soil OC (SOC), of which the belowground pools are the focus in this study (e.g. Penk & Perrin, 2022). Global estimates of SOC content in salt marshes have been attempted in previous studies, for example, Maxwell et al. (2023) who modelled global estimates from existing data, with large uncertainties such as 1220 (± 200) Mt for salt marshes to 1 m depth (Table 1). Mason et al.’s (2023) review also estimated a large range of 1410–2440 Mt (431 studies, averaging only 4.8 samples per study). The above global-scale review papers reported using OC densities from various methods within the same data set, from loss-on-ignition (LOI) analysis, digestion methods and elemental analysers (Chmura et al., 2003) or did not specify the methods used. OC stock estimates for Great Britain were calculated at 2.32 (± 0.47) Mt (0–10 cm, Smeaton et al., 2022a). The authors measured belowground SOC (no removal of roots) in an elemental analyser and incorporated secondary datasets from LOI into the main dataset by utilising their own conversion factor between total OM (TOM, LOI) and SOC (elemental analysis, EA) to derive SOC. In Ireland, Penk (2019) estimated national belowground SOC stocks for Irish salt marshes (69.3 km^2^ area), collating existing datasets to estimate from 2.59 to 4.91 kg C m^−2^ for the top 10 cm of soil. At a regional scale, Burke et al. (2022) estimated carbon density (CD) for young salt marshes in Dublin (4 sites, 1 m depth), at 11.2 kg C_org_ m^−2^ of which 87% was SOC (Table 1). In the Rogerstown estuary, CD (per m^2^) was the highest of the four sites (Malahide, Bull Island, Baldoyle) at 16.8 kg C_org_ m^−2^. OC stocks are noted to be under- or overestimated due to factors such as varying soil depths in salt marshes and a lack of global field data and measurement consistency (e.g. Maxwell et al., 2024; Smeaton et al., 2023). Both Irish studies utilised LOI and the Craft et al. (1991) conversion for belowground SOC%. In the above review, we see that OC stock estimates are based on very few samples and extrapolated to regional and global estimates.

**Table 1:** Examples of salt marsh carbon measurements in previous studies

Location	Site	Carbon Stocks / Content	Common Units	Carbon Accumulation Rate	Common Units	Sites	Notes	Reference
Global	Salt marsh review			0.21 ± 0.02	kg m^-2^ yr^-1^	24 studies	Review	Chmura et al., 2003
Global	Salt marsh surface 0.5 m	430,000,000 ± 30,000,000	t			24 studies	Review	Chmura et al., 2003
Global Average	Salt marsh			0.242 ± 0.026	kg m^-2^ yr^-1^	143 sites	Review	Ouyang & Lee, 2014
Global Average	Northern Europe SM			0.315 ± 0.063	kg m^-2^ yr^-1^	143 sites	Review	Ouyang & Lee, 2014
Global	Salt marsh 1m	1,220,000,000 ± 200,000,000	t			99 studies	Review	Maxwell et al., 2023
Global	Salt marsh 0–30 cm	7.92 ± 3.81	kg m^-2^			99 studies	Review	Maxwell et al., 2023
Global	Salt marsh 1 m	23.1 ± 13.4	kg m^-2^			99 studies	Review	Maxwell et al., 2023
Global	Salt marsh 1 m	1,440,000,000	t			3710 training locations	Review, Model	Maxwell et al., 2024
Global	Salt marsh 1 m	1,410,000,000 - 2,440,000,000	t			431 studies	Review	Mason et al., 2023
Great Britain	Salt marsh 0–10 cm	2,320,000 ± 470,000	t			752 samples, 438 marshes	Field	Smeaton et al., 2022a
Great Britain	Salt marsh 0–10 cm	5.14 ± 1.04	kg m^-2^				Field	Smeaton et al., 2022a
England	Salt marsh 0–10 cm	1,601,000 ± 426,000	t				Field	Smeaton et al., 2022a
England	Salt marsh 0–10 cm	4.5 ± 1.2	kg m^-2^				Field	Smeaton et al., 2022a
Scotland	Salt marsh 0–10 cm	368,000 ± 91,000	t				Field	Smeaton et al., 2022a
Scotland	Salt marsh 0–10 cm	6.31 ± 1.56	kg m^-2^				Field	Smeaton et al., 2022a
Wales	Salt marsh 0–10 cm	351,000 ± 82,000	t				Field	Smeaton et al., 2022a
Wales	Salt marsh 0–10 cm	6.1 ± 1.43	kg m^-2^				Field	Smeaton et al., 2022a
Great Britain	Salt marsh 1 m	9,774,000 ± 1,006,000	t			448 sites derived from 26 marshes	Field	Smeaton et al., 2023
Great Britain	Salt marsh			0.111 ± 0.043	kg m^-2^ yr^-1^	21 marshes	Field	Smeaton et al., 2024
Australia, Eyre Peninsula	Salt marsh 0–50 cm	7.01 ± 3.41	kg m^-2^				Field	Russell et al., 2024
Ireland	Salt marsh 0–10 cm	2.59 to 4.91	kg m^-2^			246 plots, 15 marshes	Field, Review	Penk, 2019
Dublin	Salt marsh 1 m	11.21 ± 1.01	kg m^-2^			4 sites, 8–12 plots	Field	Burke et al., 2022
Rogerstown estuary	Salt marsh 1 m	16.8 ± 1.79 standard error	kg m^-2^			1 site, 8–12 plots	Field	Burke et al., 2022
Schienmonnikoog, Netherlands	Back barrier SM 45 yrs old	3.3	kg m^-2^				Measured TOC	Elschot et al., 2015
Schienmonnikoog, Netherlands	Back barrier SM 35 yrs old			0.126 ± 0.009	kg m^-2^ yr^-1^		Field	Elschot et al., 2015
Tollesbury, Essex	Restored SM 0–20 yrs	2.15	kg m^-2^	0.104	kg m^-2^ yr^-1^	3 samples	Model, Field	Burden et al., 2019
Tollesbury, Essex	Restored SM 20–50 yrs	4.07	kg m^-2^	0.064	kg m^-2^ yr^-1^	3 samples	Model, Field	Burden et al., 2019
Tollesbury, Essex	Restored SM 50–100 yrs	7.34	kg m^-2^	0.065	kg m^-2^ yr^-1^	3 samples	Model, Field	Burden et al., 2019
Tollesbury, Essex	Natural 0–30 cm	6.9 ± 1.4	kg m^-2^			9 samples	Model, Field	Burden et al., 2019
Tollesbury, Essex	Restored 0–30 cm	5.9 ± 1.0	kg m^-2^			4 samples	Model, Field	Burden et al., 2019
South Korea	Natural	19.8	kg m^-2^				Model soil C	Byun et al., 2019
South Korea	Restored	14.6	kg m^-2^				Model soil C	Byun et al., 2019
E. Australia Subtropical estuarine	Salt marsh 0–3 m	82.3 ± 13.8	kg m^-2^				Field, Mean	Cacho et al., 2021
E. Australia Subtropical estuarine	Boambee Creek downstream	1.34	%				Field	Cacho et al., 2021
E. Australia Subtropical estuarine	Boambee Creek downstream	16.36 ± 7.59	kg m^-2^				Field	Cacho et al., 2021
E. Australia Subtropical estuarine	Boambee Creek upstream	2.85	%				Field	Cacho et al., 2021
E. Australia Subtropical estuarine	Boambee Creek upstream	152.56 ± 32.74	kg m^-2^				Field	Cacho et al., 2021

Local variability is important to consider, to establish the full site-wide OC stock estimate, as several studies have highlighted high variability in local OC stocks and uncertainty in up-scaled marsh-wide OC stocks (Austin et al., 2021; Supplementary material Table 1). Austin et al. (2021) estimated 367,888 ± 102,278 t OC in Scottish marshes (243) in the top 10 cm across various vegetation types (i.e. an uncertainty of ± 27.8% in this average estimate for Scotland). They also calculated a CD of 6.0 ± 1.8 kg m^−2^ (i.e. an uncertainty of ± 30%). Austin et al. (2021) demonstrated the impact of estimating UK salt marsh OC content without consideration of local variability. Downscaling from Luisetti et al.’s (2019) value, at 13 Mt OC, they estimated Scottish OC stocks at 1.72 Mt of OC by salt marsh area, which is significantly higher than existing national estimates. For example, compared to Beaumont et al. (2014)’s value, Luisetti et al.’s (2019) value overestimates Scottish OC by a factor of 3.1, and by Austin et al.’s (2021) own carbon stock estimate, Scottish OC is overestimated by a factor of between 3.6 and 6.4. Due to the uncertainty demonstrated just for Scotland, the figures demonstrated for the UK are likely to lie within a much larger range than the one average figure, e.g. provided in Luisetti et al. (2019). The importance of sample location and timing impacting belowground SOC storage was highlighted in a US study, which found 63% variation in SOC% (total) between their sites with differing biogeochemical and hydrological factors, however with very limited samples (only 3 cores at each of 3 subsites; Fettrow et al., 2024). Site-specific estimates are thus important, taking into consideration the spatiotemporal variation in SOC.

From the above review, several studies investigate specific controls individually; however, the question remains as to how these factors interact locally within a marsh system. The cumulative effect of all controlling factors on fine-scaled (within-system) variability of SOC and CD distribution, alongside the respective distributions of known important factors (AGB, BGB, elevation, sedimentary), are relatively unknown. Secondly, the review of the various factors mentioned above and the complex interactions between them demonstrate the high variability in OC content within salt marsh systems. There is an absence of salt marsh–wide (single-system) studies with sufficient samples to validate whole-system salt marsh OC stock estimates, leading to uncertainty in regionally and globally upscaled OC stock estimates.

In our study, we set out to better understand this variability at the scale of an individual salt marsh system using a higher resolution and higher density of samples than existing studies. The first aim of our study was to determine and quantify within-marsh (10 s–100 s m scale) spatial variability in SOC (i.e. as % carbon by weight) and CD (weight by volume), alongside the spatial distribution of important environmental factors (AGB, BGB, elevation, sedimentary). Hereafter, SOC is defined as a consistent measure of a stable fraction of belowground OC unless specified. The second aim was to explore the implications of patterns in the spatial distribution of CD and environmental factors for the determination of marsh-wide CD estimates using a lower density of samples. One key challenge is to determine the minimum sample number that would provide a mean which is not statistically different from the site-wide CD (*n* = 60) and to determine the deviation of the CD established from subsamples (*n* = 5, 10, 15 and 20), from the site-wide CD estimate obtained from various sampling strategies.

## Study area

**Fig. 2:**
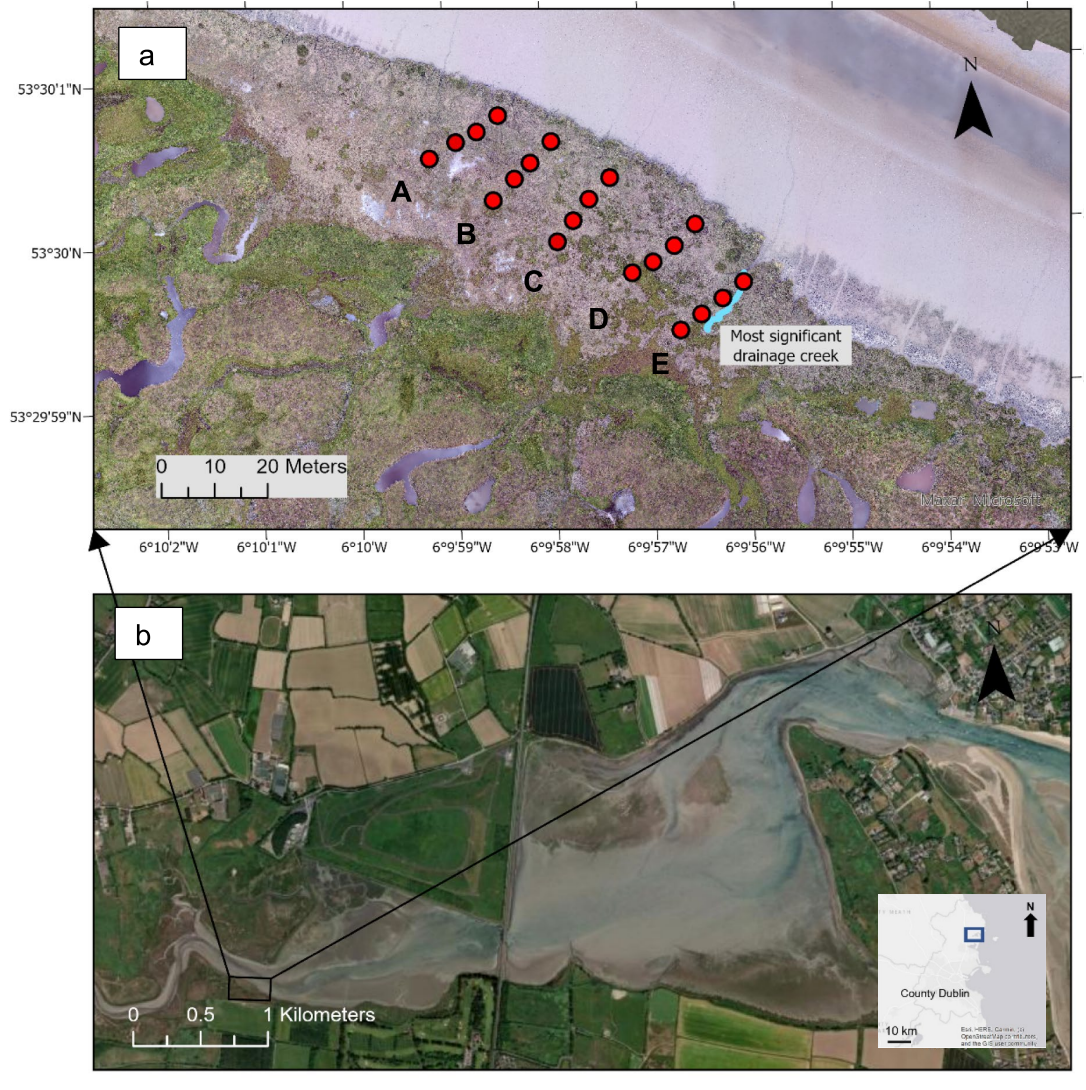
Study area, Turvey Nature Reserve, Rogerstown: **a** field sample sites in Turvey Nature Reserve; **b** Rogerstown estuary indicating the location of Turvey Nature Reserve, with an inset of county Dublin showing the general study area of Rogerstown (heritagemaps.ie). Bold letters A–E = sampling transects

The study site is a salt marsh in Turvey Nature Reserve, located in the inner bar-built Rogerstown estuary, near Donabate, Co. Dublin (Fig. 2). The estuary spans 3.68 km^2^ and is divided from the Irish sea by a sand spit, the Portrane peninsula to the south and Rush to the north (McManus et al., 1991). The sediment source for bar-built estuaries along the east coast was glacial bluff erosion, consisting of sandy mud and mixed sediment (Carter et al., 1984; NPWS, 2013). The tidal regime is mesotidal (2–4 m; Cooper, 2006), and the dominant wind direction across the marsh is WSW. Due to a wide range of habitats, parts of the Rogerstown estuary are designated as a Special Area of Conservation and Special Protection Area. Freshwater input is primarily from the Ballyboghil and Ballough rivers into the western section, while freshwater also comes from eastern channels or streams, alongside the tidal inundation of marine water. In the 1840 s, during the development of the Dublin to Belfast railway, a causeway and bridge were built across the estuary somewhat restricting tidal influx and impacting the drainage between the inner and outer estuary through weakened ebb and flood currents (Fahy et al., 1975; Mulrennan, 1993; Calder, 2020). As such, the salt marsh in the inner estuary experiences inundation primarily during high spring tides (e.g. NPWS, 2013), but as measured during a field visit on 12/03/2024 during the equinox, on such occasions, water depths can reach up to 0.8 m over the marsh platform. To place the marsh in context in the regional and international literature, the inner estuary salinity (in PSU) measured by Kerr et al. (2023) was 30.1 (high tide) and 20.1 (low tide). For the outer estuary, salinity was 30.9 (high tide) and 28.4 (low tide).

The salt marsh at Turvey Nature Reserve is constrained by the Ballough river on the north and agricultural and grazed land lying landward and to the south. The marsh surface consists of 1.1–1.5 m of coastal clay above a 4–15-cm-thick organic peaty deposit, with a deep clay layer below the peat (Fingal County Council, 2022). In the wider area, around 0.7 km inland from the study site, the site is being actively managed by Fingal County Council to restore the natural hydrology throughout the reserve. Constrained between the marsh edge at the river and a grassland bank around 35 m back from the marsh edge, the marsh is water-logged in some areas; however, drainage is relatively uniform across the marsh site. Based on the elevation measurements conducted in this study (see “Field and laboratory methods”), the average elevation is 1.73 m (Malin Head Datum, approximating mean sea level, October 2022). In this study, the largest small natural creek (hereafter “the creek”, ~ 0.2-m width and 12.9-m length of the channel visible from the marsh surface, southward from the channel mouth (narrow edge) entering the marsh to the back of the marsh) was utilised as a boundary to investigate sediment sampling strategies and was located to the east of transect E (see methods; Fig. 2). Plant species include dominant mid- and low-marsh zone species, *Spartina anglica*, *Atriplex portulacoides* and *Puccinellia maritima* areas. *Triglochin* sp. and *Armeria maritima* are also present (e.g. Perrin et al., 2020). The woody plants (*Atriplex portulacoides*) were primarily located along and within 5 m of the marsh margin and along the creek. Elsewhere across the site was dominated by herbaceous species (grasses). For comparison to other marshes, the pH measured during this study (using the “Hanna” multimeter) in the creek was 6.85 and soil pH (“ETI 8000” instrument) was 6.7, with a soil conductivity (“EcoTestr11” multimeter) of 9.8 mS (taken west of transect C (see Methods) in the middle of the site, June 2024).

## Field and laboratory methods

### Field sampling grid

**Fig. 3:**
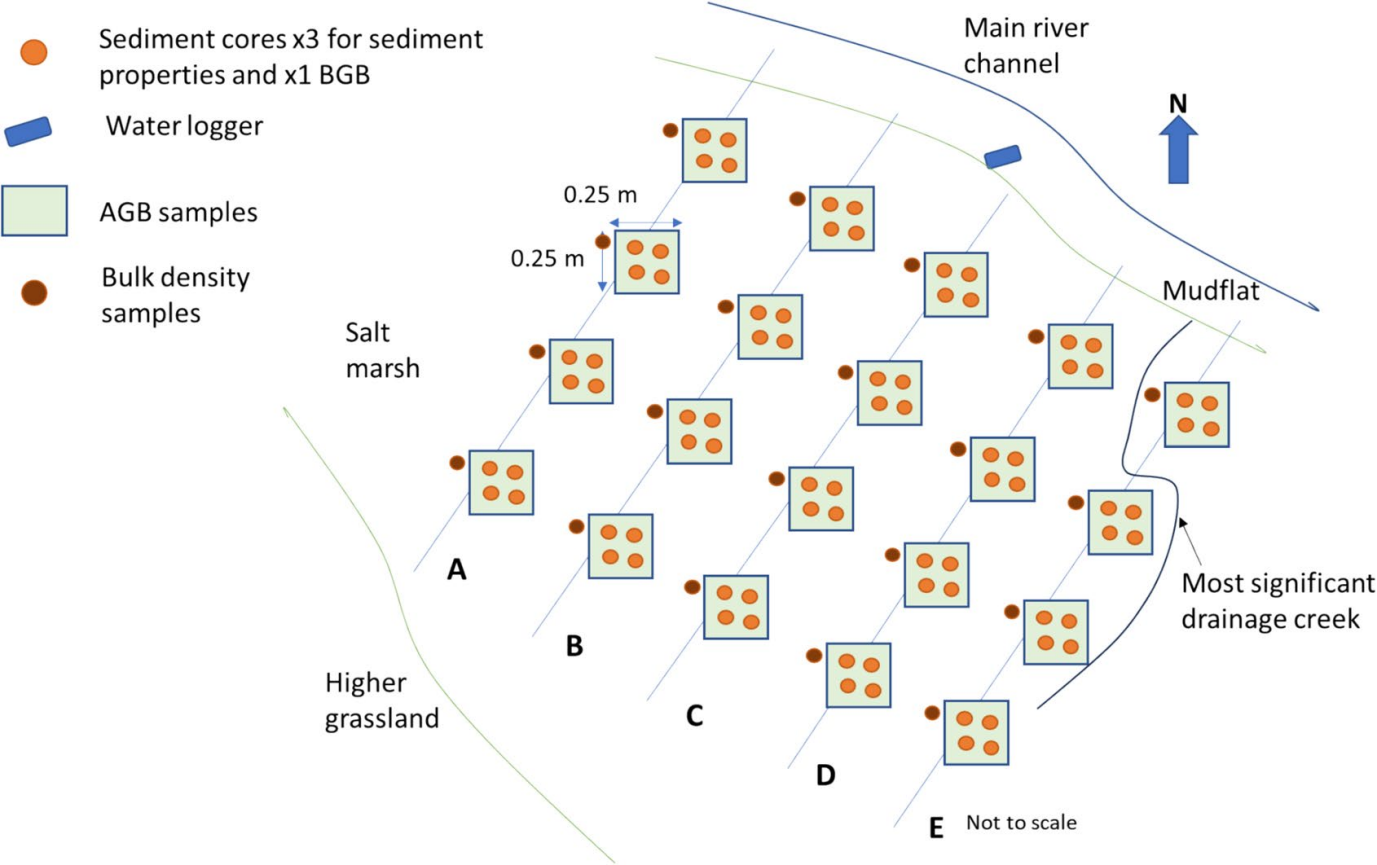
Sampling layout

To capture differences in surface elevation, vegetation and hydrological connectivity (distance from the marsh margin) alongside SOC storage, 20 soil and vegetation sampling sites were distributed across the salt marsh site in a semi-structured grid pattern (Figs. 2 and 3). The sampling grid was laid out following Owers et al.’s (2022) recommendation of stratified sampling to allow for identification of specific location controls not due to random sampling. Five transects (A–E) were laid out perpendicular to the main river channel, spaced approximately 10 m apart along the river. Twenty quadrats defining each sampling site, within which vegetation and soil samples were taken, extended towards the back of the marsh, 4 per transect at 5-m intervals (hereafter referred to as, for example, A5 = transect A, 5 m distance from marsh margin). Position and elevations for each sampling site were recorded using an R8s Trimble differential global positioning system to a vertical accuracy of ≤ 1.5 cm (Malin Head Datum; Trimble, 2017).

### Aboveground biomass sampling

To assess spatial biomass variability, AGB samples were taken on 10th–11th August 2023 from a 25 cm^2^ quadrat at each sampling site (*n* = 20, Fig. 3), with the top left corner of the quadrat orientated north and ensuring that only plants with roots originating within the quadrat boundary were included. Vegetation was cut to the sediment surface using secateurs, and samples were stored in sample bags at 4 °C prior to analysis (following Burden et al., 2019; Penk et al., 2020).

In the laboratory, AGB samples (*n* = 20) were separated into species, whilst algae, plant litter and non-plant material were removed. The separated subsamples were oven-dried at 65 °C for ~ 48 h (max 72 h) and weighed to 2 d.p., then rounded to the nearest 0.1 g (following Penk et al., 2020). Species were then categorised as “herbaceous” (e.g. grasses) or “woody” (e.g. *Atriplex* spp.), to highlight areas of similar plant structures, and the mass per category was summed. For quality control, all laboratory analysis was carried out on the same scales and equipment washed before and between usage.

### Sediment sampling

#### Cores

Sediment cores (*n* = 80) were obtained using a Russian corer (7.5 cm diameter, 50.8 cm depth, following Smeaton et al., 2020) between 24th August and 5th September 2023, with four cores taken from each 25 cm^2^ quadrat used for AGB sampling. The use of a Russian corer ensured that compaction was minimal or absent during the extraction process (Smeaton et al., 2020). Three cores were utilised for sediment properties, and the fourth was reserved for BGB (e.g. following Burden et al., 2019). The length was measured in the field and recorded to assess compaction during transport. Following measurement in the laboratory, we established that no cores had compacted (< 0.1 cm change). Upon return from the field, cores wrapped in clingfilm and tin foil and labelled in the field were stored in the fridge at 4 °C. Samples were moved to the freezer at − 27 °C from 08 Dec 23 to 18 Jan 24 to reduce organic activity prior to LOI analysis. To ensure freezer storage did not have a significant impact on soil parameters reported in this study, separate subsamples from the cores used for BGB were homogenised and split between fridge and freezer storage for the same period as the main frozen samples (*n* = 20). LOI was then conducted on each subsample using the same method as the main dataset (“Sediment properties”). *R*^2^ was calculated between the fridge and freezer datasets for four key LOI measurements which were utilised for soil parameter calculations (data not shown). High correlation coefficients were obtained for moisture content % (MC; *R*^2^ = 0.89), LOI (g) at 480 °C (*R*^2^ = 0.83), LOI (g) at 400 °C (*R*^2^ = 0.83) and CaCO_3_% (*R*^2^ = 0.87) and were significant at *p* < 0.05.

#### Core subsampling

Cores for LOI were subsampled into two samples in 2-cm-width sections at 10 cm (from 9 to 11 cm) and 30-cm depth (from 29 to 31 cm) with a ceramic knife. Each wet sample was then homogenised using a pestle and mortar to ensure an even distribution of SOC within the subsample (following Howard et al., 2014), then stored in sample bags in the fridge at 4 °C. All sub-sampling was carried out within the surface coastal clay layer (Fingal County Council, 2022), and sampling at 10 cm and 30 cm provided a depth comparison within the root zone for belowground OC (Houston et al., 2024).

#### Belowground biomass

Cores for BGB determination were measured and photographed before being subsampled to 30-cm depth (following Penk et al., 2020), and the roots visible on the outside were cut off to include only BGB originating from within the sample. Roots were washed over a 2-mm and 1-mm sieve stack before being sorted into live (white, partially grey/brown and round diameter or attached to white) and dead (black, flattened diameter and grey/brown) roots (Reef et al., 2017). BGB samples (*n* = 20) were dried at 65 °C for 48 h or until constant weight was achieved and then weighed to 2 d.p. (per 44.2 cm^2^ area to 30-cm depth; following Burke et al., 2022; Penk et al., 2020; Penk & Perrin, 2022).

### Sediment properties

#### Loss-on-ignition

LOI was carried out to obtain physical parameters for the cores following a version of the sequential Cambridge protocol, modified to obtain SOC in one analysis sequence by the lead author (University of Cambridge, 2022; Supplementary material 2). There are various methods in the literature for measuring SOC (see below and “Spatial distribution of carbon and environmental factors”); thus, our modified LOI method was utilised as a site-specific alternative to the Craft (1991) conversion (e.g. Burke et al., 2022), which can cause overestimation (Smeaton et al., 2022a) and in case access to EA is unavailable. Our EA results and the conversion factor from TOM are also provided to facilitate methodological comparison. The protocol was adapted by drying 2 cm^3^ samples at 65 °C, then heating sequentially at 400 °C, 480 °C and 950 °C for 6 h at each temperature increment in a furnace. The outputs are MC (%, Eq. 1) following oven drying, SOC (%, Eq. 4, from which CD was calculated, see “Bulk density”), TOM (%, Eq. 2) following 480 °C and calcium carbonate (CaCO_3_%, Eq. 3) following 950 °C. Samples were cooled in a desiccator before weighing to 4 d.p. and calculating each sediment parameter.

There are various methods used for LOI (Dean, 1974; Salehi et al., 2011), with differing temperatures, leading to investigations of the suitable temperature range for the full oxidation of OM. This study focuses on spatial variation of TOM and SOC; therefore, the cheaper and more accessible LOI methodology is suitable to highlight relative site-wide changes in TOM and SOC. A comparison to OC derived from EA was also conducted to create a conversion factor (see below). Studies have demonstrated that the accuracy of TOM (including SOC) measurement from LOI is greater at lower temperatures, from ~ 360 to 450 °C, as this eliminates or significantly decreases structural water loss in clays and is unaffected by CaCO_3_ content (Ball, 1964; Davies, 1974; Howard et al., 2014; Salehi et al., 2011). High temperatures, such as > 550 °C have also been indicated to damage structural integrity of the samples and cause overestimation due to additional mass loss (Davies, 1974; Lebron et al., 2024); however, presence of CaCO_3_ did not impact LOI at 430 °C (Davies, 1974). Thus, 480 °C for TOM (including SOC, roots) balance preservation of the clay structure and loss of carbonates while still oxidising TOM. Furthermore, studies have indicated two key thermal mass loss peaks, representing two organic fractions, which support the separation of TOM and SOC fractions during analysis (Lebron et al., 2024; Schnitzer & Hoffmann, 1966). Thermodynamically stable SOC does not burn at < 375 °C and starts oxidising above 400 °C; thus, the lower temperature (400 °C) provides a TOM measurement without the stable SOC fraction (Lebron et al., 2024). For CaCO_3_ determination, studies suggest carbonates are removed at temperatures ranging from 850 to 1000 °C (Dean, 1974; Fu et al., 2020; Heiri et al., 2001; Veres, 2002). Additional oxidation of OC, however, could increase the total inorganic carbon (TIC) measurements from LOI; thus, EA has also been provided. Carbonates may be lost at lower temperatures; however, carbonates have been found to decompose above 700 °C, which is higher than the temperature for TOM in this study (Davies, 1974; Lebron et al., 2024). Additionally, a caveat of this technique is the potential loss of other minerals, such as carbonates containing Mg, Ca or Fe, which may increase SOC measurements (e.g. Shamrikova et al., 2023).

Water logging increases decomposition rate; therefore, MC was assessed as an indication of retention potential of carbon (e.g. Hemminga et al., 1991). The MC (Eq. 1), represents pore water content, calculated as mass lost after oven-drying. TOM, representing mass lost post-heating as a percentage of initial dry weight (Eq. 2; e.g. Ball, 1964; Davies, 1974), was utilised to calculate SOC. CaCO_3_, as a percentage of initial dry weight, was calculated via a conversion factor using a ratio of the molecular weights of CaCO_3_ to CO_2_ lost (Eq. 3, University of Cambridge, 2022). SOC calculated as a percentage of initial dry weight represents the mass lost between ash residue at 400 °C and 480 °C to distinguish between TOM and the SOC fraction (Eq. 4):

1 $$MC\;(\%)=\;(FS-OD)/FS\times100$$

where FS = fresh sample (g), before drying, and OD = oven dried at 65 °C (g).

2 $$TOM\;(\%)=\;(OD-AR480)/OD\times100$$

where ARX = ash residue at X °C.

3 $$CaCO3\;(\%)\;=\;((AR480-AR950)\;\ast\;2.274)/OD\times100$$

4 $$SOC\left(\%\right)=\left(AR400-AR480\right)/OD\times100$$

#### Bulk density

Bulk density (BD) samples were taken between 5th September and 2nd October 2023 (*n* = 60) to assess the horizontal spatial variability in CD, calculated using the measured BD. To reduce disturbance common when applying standard cubic centimetre sampling devices to highly organic/root-rich salt marsh sediments, a wedge was cut from the sediment surface, 5–10 cm outside the top left corner of each of the 20 quadrats, using a trowel (west of the cane; Supplementary material Fig. 1a). The BD sample was then subsampled between 5- and 10-cm depth using a penknife to cut ~ 1-cm-width wedges from the relatively undisturbed edge of the removed sediment, with 3 replicates to obtain a mean value in the laboratory (Supplementary material Fig. 1b-d). It should be noted that the depth of the BD samples relates most closely to 10-cm samples; spatial comparison is made in this study primarily to samples at 10 cm; however for CD, the calculations at more compacted 30-cm-depth layers should be considered with caution. The samples (a, b, c) were carefully and gently wrapped in clingfilm to reduce disturbance, placed in a small sample bag and then into a plastic box with individual samples separated with tissue (Supplementary material Fig. 1e). This ensured samples were not crushed or able to move during transport and storage. The boxes were transported in a larger crate from the field to campus and placed in cold storage.

In the laboratory, to ensure that sampled sediment volumes from the field used for BD estimation represented assumed volumes accurately, each BD sample (a, b, c) was weighed to 4 d.p., then wrapped carefully in clingfilm, ensuring there was no compression and no excess film (Supplementary material Fig. 1f-h). Volume was then measured, by placing the sample in a known volume of water in a cylinder with 1-ml increments. The change in volume was measured to the nearest 0.5 cm^3^ once the sample was added (Supplementary material Fig. 1i). The samples were then unwrapped and dried at 50 °C for up to 48 h (until completely dried, following Smeaton et al., 2020). The dry weight was measured for dry BD (DBD) to 4 d.p. on the same scales, dividing the mass by the measured volume (g cm^−3^). A known volume (2 cm^3^) from a volumetric sampler was utilised to assess error, and 2 cm^3^ was achieved, confirming that the method was accurate. CD was calculated by multiplying SOC% by DBD.

For comparison of our method to existing regional-scale estimates and to place our Rogerstown estuary results in context with Irish estimates, mean CD (kg m^−3^) was upscaled to Irish salt marsh area (Penk, 2019) by converting from density to area, assuming a depth of 10 cm to convert to stocks (t C stocks). Both values represent belowground CD.

#### Elemental analysis

**Fig. 4:**
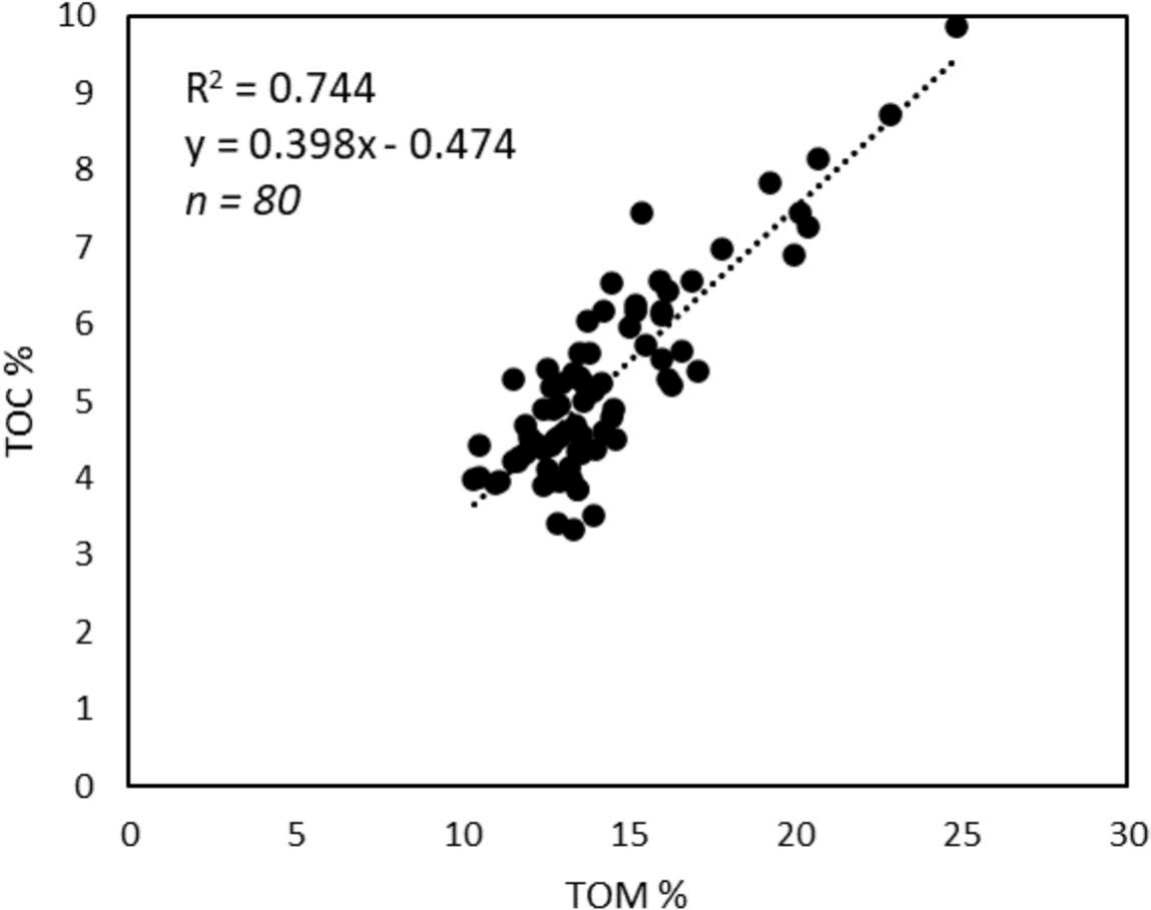
Total organic matter content from loss-on-ignition compared to total belowground organic carbon from elemental analysis (*R*^2^ = 0.74; *p* < 0.01)

Subsamples from 10- and 30-cm core subsamples, including roots (~ 1–2 g) were oven-dried for > 12 h at 105 °C (until completely dried) and ground using a pestle and mortar (following Smeaton et al., 2022a; Vereş, 2002). Belowground carbon content was obtained using a Multi EA4000 elemental analyser, which measured to 10% accuracy in relation to CaCO_3_ standard measurements throughout for quality control. The differential method was utilised to measure the total carbon (TC) and TIC, deriving total organic carbon (TOC) from the difference between TC and TIC. This method used acidification (hydrochloric acid, HCl) to generate CO_2_, which is then measured by the NDIR detector for TIC. TC was measured separately by combustion at 1200 °C (Liu et al., 2022; Nieuwenhuize et al., 1994). In this study, to compare between LOI and EA methods, TOC% from elemental carbon analysis correlated with TOM% from LOI (*R*^2^ = 0.74, *p* < 0.01; Fig. 4). A conversion factor for this Irish study site was obtained as TOC (%) = 0.398 × OM (%) − 0.474.

#### Particle size analysis

Subsamples from the sediment cores were prepped for particle size analysis by removing OM and carbonates, using hydrogen peroxide (H_2_0_2_) 30% solution and 20 ml HCl 10% respectively (August–September 2024; Bartminski et al., 2022; Eshel et al., 2004; Gray et al., 2010; Sperazza et al., 2004; Supplementary material 3). De-flocculant (20 ml 50 g/l sodium hexametaphosphate, Na(PO_3_)_6_ solution) was added overnight, and samples were sieved before analysis by laser particle diffraction in the Mastersizer 3000 after oven-drying at 50 °C for 48 h.

### Statistical analysis

Anderson–Darling normality tests (D’Agostino and Stephens, 1986) were conducted at the 95% confidence interval to assess the distribution of various carbon content measurements (TOC, TC, SOC, CD).

Subsamples from the CD in this study (*n* = 60) were randomly taken using the Matlab Twister random number generator for *n* = 5, 10, 15 and 20 (30 subsamples each) to simulate various sampling strategies. Five sampling strategies were tested: Randomly subsampling all data (named rand20); randomly subsampling data plus five samples at 5 m from the marsh edge (rand20 5 m); random subsamples from within 20 m of the creek (taken from transects D and E, see “Study area”, rand20 creek); random subsamples from within 5 m of the marsh margin (rand15 margin) and random subsamples > 20 m from the creek and > 10 m from the marsh margin (rand20 mid). The 30 subsamples were then each compared to the site-wide full-field dataset across the 60 samples (hereafter referred to as the ‘site-wide CD’) using the two-sample *t* test, assuming equal variances (95% confidence level) to establish whether CD mean from each subsample (hereafter ‘subsample CD mean’) and the site-wide CD mean were not significantly different (came from independent random samples from a normal distribution with equal means). The central limit theorem was applied to use the *t* test for a large dataset *n* = 60, and the subsamples were from the parent dataset. The difference in means between subsamples and site-wide CD in this study and the spread of *p* values from the *t* test were utilised to analyse the probability of obtaining a subsample CD mean from various sampling strategies that was not statistically different (95% confidence) to the measured site-wide CD mean in this study. Extreme maximum values have not been discussed here, due to the possibility that they may be unlikely outliers.

Kernel density was carried out on CD and environmental factors (e.g. AGB, BGB, TOM) in ArcPro to assess spatial distribution, using the Spatial Analyst toolbox, with the planar setting for local-scale analysis and a small search radius (0.00005) to achieve the best visual contrast between sites for a small site area (Silverman, 1986).

## Results

### Spatial distribution of soil organic carbon, carbon density and environmental factors

**Fig. 5:**
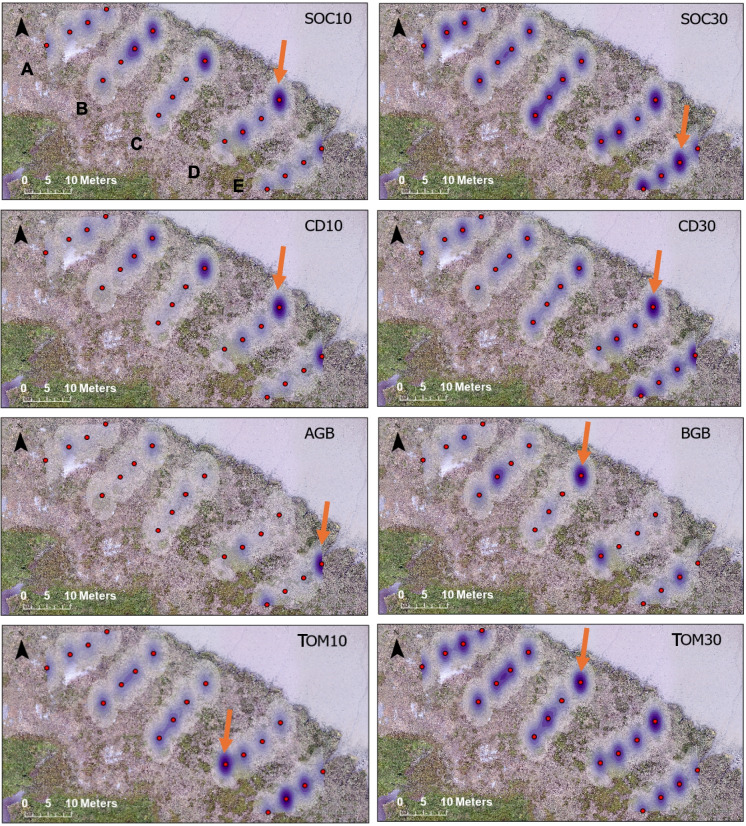
Spatial variation in SOC and CD alongside other parameters across the site. SOC at 10-cm and 30-cm depth; CD at 10-cm and 30-cm depth; AGB, BGB; TOM at 10- and 30-cm depth. The colour shade represents the kernel density value per unit area for the cell. A darker colour thus represents a higher density area for the parameter, truncated at edge of the site area where data was not extrapolated. Red arrows = highest value

The spatial distribution in SOC, CD and environmental factors (AGB, BGB, TOM) across the marsh was visualised via kernel density heatplots (Fig. 5). At 10-cm depth, SOC was highest along the marsh margin (3.5%). The CD revealed a similar pattern at 10 cm, also highest at the marsh margin (19.8 kg m^−3^), but it varied along transects with increasing distance from the creek. The same distribution is not reflected in AGB or BGB; however, peak biomass did occur near the marsh margins. The highest AGB occurred within centimetres of the creek (1.82 kg m^−2^), and BGB was highest at 5 m from the marsh margin (Fig. 3, 2.37 kg m^−2^ to 30 cm depth). The TOM peaked at 15–20 m from the margin, within 5 m from the creek (22%). Between 10- and 30-cm depth, the apparent margin control for SOC disappears. From 10 to 30 cm, SOC increases to a more even distribution across the site, however, CD is more similar with depth. Similarly to SOC, TOM is higher at depth than at the surface. For comparison of our methodology to existing national-scale estimates and to place our results for the Rogerstown estuary in context with existing Irish estimates, the upscaled mean CD for 10 cm depth from this study for Irish salt marshes (69.3 km^2^, Penk, 2019) is 76,923 t C.

### Variation of carbon and environmental factors with distance from marsh margin

**Fig. 6:**
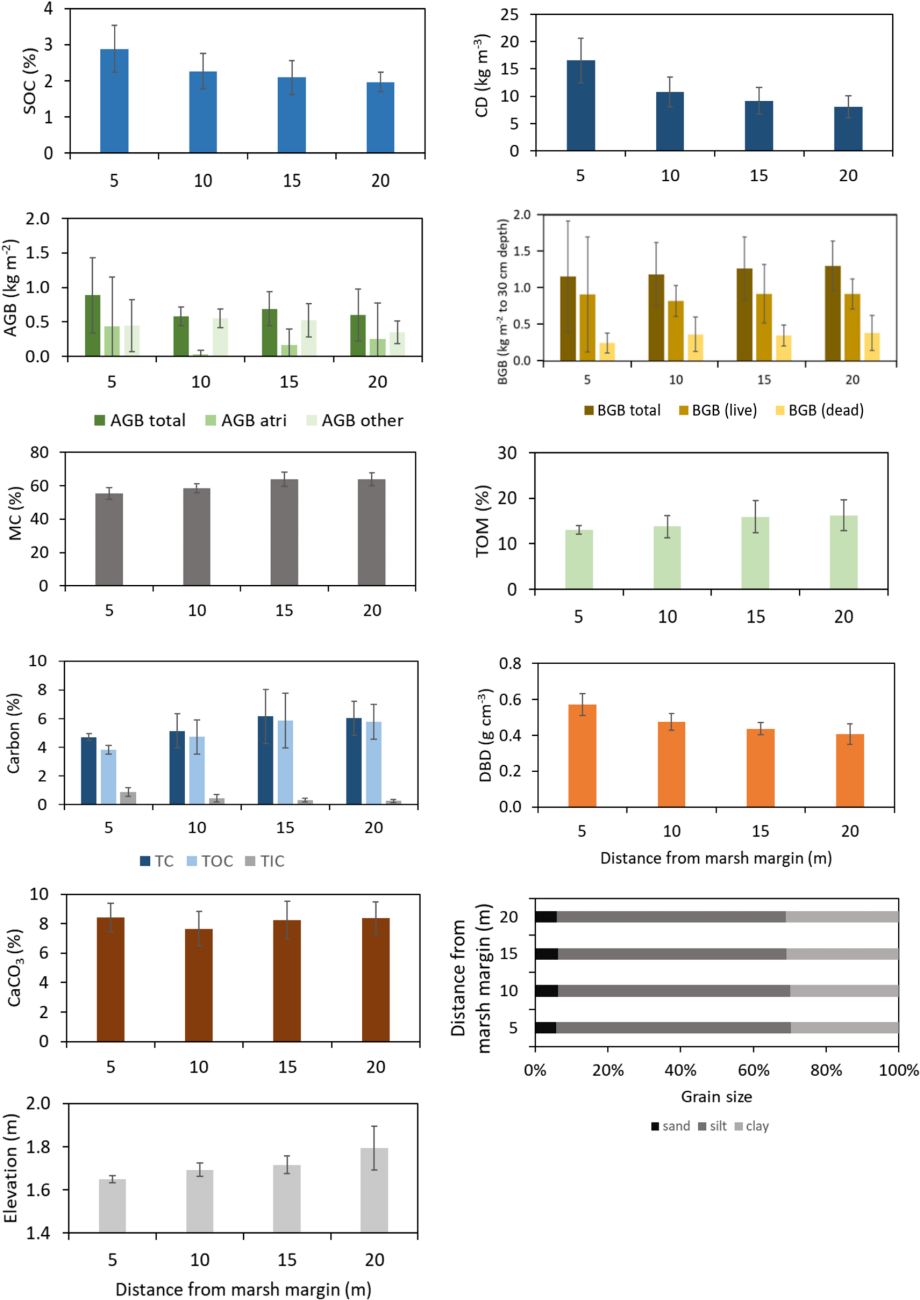
Variation of sedimentary and environmental characteristics at 10-cm depth and above- and belowground biomass (*y*-axes) with distance from the salt marsh margin facing the main river channel (*x*-axis). AGB: total = total aboveground biomass, atri = mass of *Atriplex* spp., other = mass of herbaceous spp. Grain size at 10-cm depth (*x*-axis) and distance from marsh margin (*y*-axis). Sand > 63 µm, silt 63–4 µm, clay < 4 µm

To investigate how OC varies across the salt marsh, averaged OC measurements (SOC, CD, TOC, TC) and various environmental factors (e.g. AGB, BGB, elevation, particle size) were investigated between 5 and 20 m from the marsh margin at 10-cm depth (Fig. 6). In SOC and CD, there is a general decrease with increasing distance from the marsh margin (by 0.9% and 8.5 kg m^−3^ respectively) alongside a decrease in DBD. The marsh margin CD was 174% larger than the marsh interior CD. The TOC and TC from EA generally increase with distance from the marsh margin (by 1.9% and 1.3% respectively), alongside MC, TOM and elevation, which becomes more variable 20 m from the marsh margin. The TOC% is higher overall than SOC%. TIC% decreases with distance from the marsh margin; however, CaCO_3_ decreases within the first 10 m. The inorganic carbon fraction in CaCO_3_ (12%) is higher than TIC%, highlighting the caution that is advised when combining datasets from mixed methods. AGB is variable for both *Atriplex* spp*.* near the margins, and herbaceous species mid-marsh with increasing distance from the marsh margin; however, BGB remains similar. At the within-marsh scale in this study, particle size varied little with distance from marsh margin from 62.8% silt (15 m) to 64.4% silt (5 m).

### Descriptive statistics

Anderson–Darling normality tests revealed non-normal distributions in elemental carbon analysis at 10 cm (TOC and TC; Table 2) and CD from LOI at 10 cm and 30 cm, highlighting the difference that arises between belowground carbon measurements.

**Table 2:** Anderson–darling tests for salt marsh carbon measurements (BPI Consulting, 2024)

Variable	Dataset	AD	*p* value	*n*
TOC 10 cm	Elemental	2.48	< *0.001*	40
TOC 30 cm	Elemental	0.49	0.207	40
TC 10 cm	Elemental	3.42	< *0.001*	40
TC 30 cm	Elemental	0.51	0.191	40
SOC 10 cm	LOI	0.54	0.163	60
SOC 30 cm	LOI	0.58	0.125	60
CD 10 cm	LOI	1.26	*0.003*	60
CD 30 cm	LOI	0.83	*0.030*	60

Italics = non-normal distribution (*p* < 0.05).

*TOC* total organic carbon; *TC* total carbon.

### Carbon density ranges

The mean CD for the site was calculated from 10-cm depth, utilising CD taken from all 20 sample grid sites (Fig. 2). Thus, the site-wide CD estimate for this study is 11.1 ± 4.2 kg m^−3^ (Fig. 6). The CD varied across vegetation species, differing hydrological conditions and distance from the creek and marsh margin (5.2–22 kg m^−3^, 423% increase). It was highest where woody plants (*Atriplex* spp.) are present near the marsh margin and lowest in areas dominated by herbaceous species, for example 20 m from the marsh margin.

The mean and range obtained from various sample sizes and sampling strategies was explored by randomly selecting and comparing ‘subsample CD’ datasets (*n* = 20, *n* = 15, *n* = 10 and *n* = 5) to the site-wide CD (full dataset) and testing the difference in means for significance (two-sample *t* test, 95% confidence, Table 3).

**Table 3:**
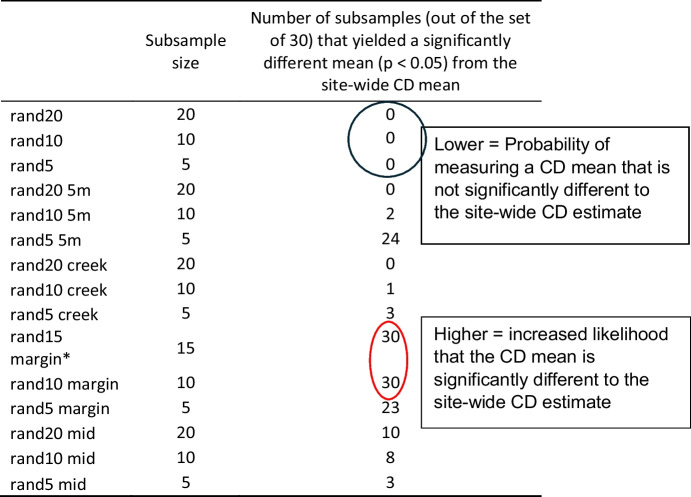
Number of subsamples with significantly different means from the site-wide mean CD estimate (full dataset *n* = 60), from the two-sample *t* test (*p* < 0.05) with the assumption of equal variance. *note: only 1 sample could be obtained for *n* = 15 at the margin instead of 30

**Fig. 7:**
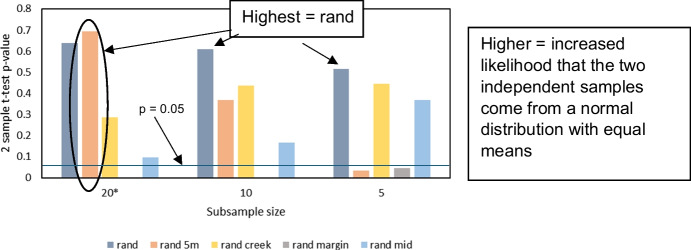
Mean *p* values from the two-sample *t* test (95% confidence) between marsh-wide site estimate for CD from the main dataset (*n* = 60) and the subsamples (*rand margin *n* = 15)

Sampling strategy impacted the likelihood of obtaining a CD mean which was not statistically different (*H*_0_ for *t* test accepted at 95% confidence) to the site-wide CD (threshold used for probability *p* < 0.05, Table 3, see precise *p* values in Fig. 7). Random sampling indicated the highest chance that the subsampled and site-wide CD mean for each sample size would not be statistically different (Table 3, blue circle; Fig. 7). Random sampling was therefore the best predictor of CD for all tested subsamples. Additionally, random samples of *n* = 5, including five samples at 5 m from the margin, was best overall (Fig. 7, black circle). Randomly sampling next to the creek is the next best method for any sample size (all subsample sizes *p* > 0.1; Fig. 7).

Sample size was also important for obtaining a mean that was not statistically different from the marsh-wide site estimate. When restricted to within 5 m of the marsh margin, 100% of 30 subsample instances for *n* = 15 and *n* = 10 produced a significantly different mean from the site-wide CD (Table 3, red circle). Similarly, when only five samples were taken, random samples still produced a mean value which was not statistically different from the site-wide CD. Subsamples of *n* = 5 provided a significantly different mean CD more than half of the time, when some or all of the samples were restricted to 5 m from the marsh edge (77% or 88% chance of statistical difference, Table 3; Fig. 7).

**Fig. 8:**
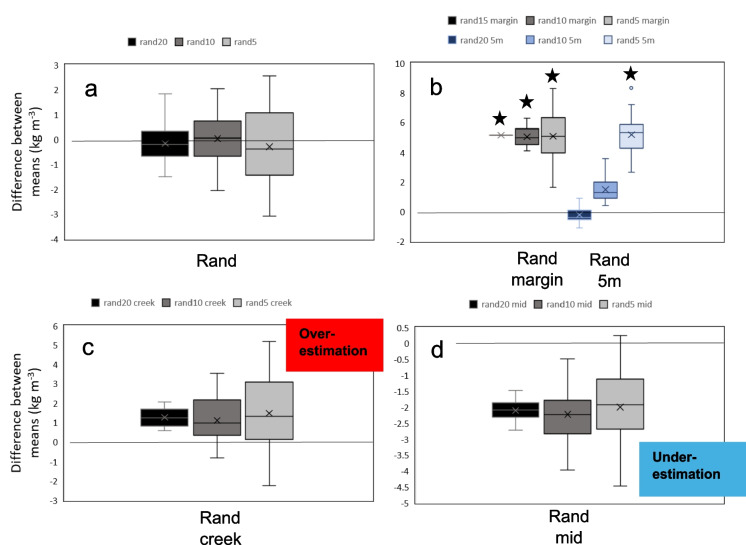
Difference between means for C density at 10 cm when subsampled (*n* = 20, 15, 10 or 5) compared to the main dataset (*n* = 60). Significant differences (*p* < 0.05) marked with a star

**Fig. 9:**
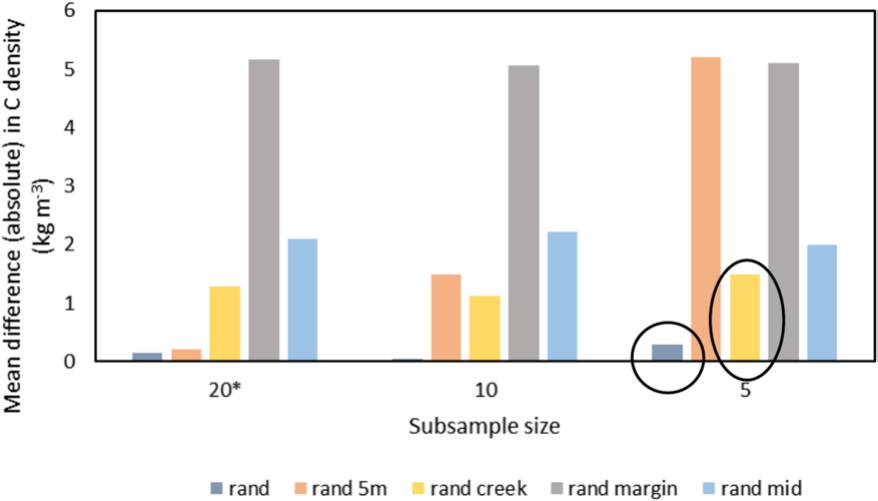
Mean absolute difference between C density values at 10 cm from the main dataset and the subsamples (*rand margin *n* = 15). Best recommended sampling strategies highlighted

The difference in CD means (site-wide and smaller subsamples) was investigated to visualise how close the subsample CD mean was to the site-wide CD estimate for various strategies and sample sizes and to demonstrate, for each strategy, the most likely outcome for the subsample CD (under- or overestimation of the site-wide CD) across 30 replicates. For random samples, estimates were evenly spread around the site-wide CD mean (mean = 11.07 kg m^−3^), mostly within 2 kg m^−3^ (18%) of the marsh-wide mean and the smallest spread was seen in the largest subsample, *n* = 20 (Fig. 8a). Random sampling also produced the lowest absolute mean difference between all subsample sizes (for *n* = 5, *n* = 10 and *n* = 20) and the marsh-wide mean, such that mean absolute difference between subsample and site-wide estimate (*n* = 60) ranged from 0.04 (*n* = 10) to 0.29 kg m^−3^ (*n* = 5) (Fig. 9).

Restriction of samples to near marsh boundaries led to an overestimation of the site-wide CD mean in most cases tested (Fig. 8b, c). When sampling within 5 m of the marsh margin, the site-wide CD mean was overestimated for all tested sample sizes (*n* = 20, *n* = 15 and *n* = 5) by a mean of 5 kg m^−3^ (a factor of 1.46 of the site-wide CD estimate). For small sample sizes of *n* = 5, such overestimation reached up to 8 kg m^−3^ (factor of 1.75; Figs. 8b and 9). Restricting samples with some near the marsh margin also led to an overestimation of the site-wide CD mean (for *n* = 15 or *n* = 20) up to a mean of 5 kg m^−3^ difference (factor of 1.47; Fig. 8b). Sampling within 20 m of the creek mostly led to an overestimation of the site-wide mean for all sample sizes; however, this strategy produced a smaller overestimation of 1.3 kg m^−3^ (factor of 1.11; Fig. 8c). Finally, samples that were away from marsh boundaries (> 20 m from the creek and > 10 m from the marsh margin) led to an underestimation of the site-wide mean by an average of 2.1 kg m^−3^ (a factor of 0.81) for all tested subsamples (*n* = 20, *n* = 10 and *n* = 5). For small sample sizes of *n* = 5, the marsh-wide mean was underestimated by up to 4.5 kg m^−3^ (a factor of 0.60; Fig. 8d). For subsamples of *n* = 5, the best sampling strategy to obtain the smallest difference from the site-wide mean was random sampling (underestimation of factor 0.98), followed by random sampling within 20 m of the creek (overestimation of factor 1.13; Fig. 9).

## Discussion

### Spatial distribution of carbon and environmental factors

In this study, we set out to better quantify spatial variability in SOC as measured through the LOI method. We found the highest near-surface SOC at the marsh margin, where the dominant vegetation is woody. Since distribution in SOC does not directly reflect AGB (Figs. 4a and 5a), this suggests a finer species composition control or habitat control (Groenendijk, 1984; Penk & Perrin, 2022). AGB distribution, in turn, which peaks at 5 m from the marsh margin and near the creek (Figs. 4a and 5a) can be influenced by tidal flooding and inundation and is demonstrated in the proxies used in previous studies of distance to the creek and marsh edge (e.g. Belknap & Kelley, 2021; Miller et al., 2023; Russell et al., 2024). Since the higher SOC near the creek in this study also does not appear to coincide with the higher BGB, it may be that the concentration of organic carbon that is captured via plants, where AGB is high, is reduced in the soil matrix through minerogenic sediment input due to proximity to the creek (e.g. French et al., 1995). High SOC at the margins could also be due to other factors such as sediment deposition from the creek and tidal influence, elevation and drainage (French et al., 1995; Moeller et al., 1999; Penk & Perrin, 2022; Reed, 1999; Schuerch et al., 2019). It is therefore important to understand these potential vegetation and geomorphological controlling factors to understand the differing influences of dominant processes on the spatial distribution of SOC burial.

Varying trends of SOC with depth between the near-surface and 30-cm depth were observed. The pattern of high SOC at the margin disappears at 30-cm depth (Fig. 4a and 5a). Conversely, the highest OM was found at the margin only at 30-cm depth, while near-surface OM peaked around 20 m from the marsh margin, as was also seen in Roner et al. (2016) and Puppin et al., (2024) for microtidal southern European marshes. They found that interactions between elevation and the variation in sediment input, and thus suspended sediment concentrations, with distance from the marsh margin influence OM distributions. Further investigation is, however, required for mesotidal environments such as in this study. Similarly to above, SOC content may be influenced by tidal inundation processes at the surface, and OM content near the creek edge could be diluted by the sediment deposition from the creek, but may be protected from such surface processes belowground, at 30-cm depth where OM is higher (e.g. French et al., 1995; Perillo et al., 2019; Reed, 1999).

CD was highest along the marsh margin for samples taken at 10 cm (mean = 16.2 kg m^−3^), by a factor of 1.73, compared to the marsh interior. This coincided with the highest DBD and dominant woody vegetation at the marsh margin (Fig. 5a). CD is more uniformly spatially distributed at 10- and 30-cm depth than SOC, since the compression is already taken into account of the sediment at depth (Fig. 4a). Allen (2000) describes the compaction of sediment during marsh accretion, which decreases the volume of sediment at depth and contributes to surface subsidence. Sedimentation causing the long-term burial can increase the SOC% at depth due to the older, anoxic sediments at depth retaining carbon for as long as the sediment remains in place (Cahoon et al., 2000). The pattern in CD is not reflected in AGB and BGB directly (Figs. 4a and 5a). Allochthonous sediment deposited on the marsh surface may be the source of OC accumulated at near-surface sediment, while plant-derived carbon from roots is more important at depth (Saintilan et al., 2013); however, this pattern is not clearly seen in the BGB vs SOC at 30 cm. The variation may be less pronounced at 30 cm due to a lowered impact from surface processes at depth (Cahoon et al., 2000).

Our additional use of EA provides a useful comparison between the methodological approaches. Values are compared here on the basis that all values represent belowground OC measurements (including roots). From EA (TC, TIC, TOC), we demonstrated different trends to the LOI measurements (SOC, CD). SOC is lower overall than TOC, since in this study, SOC represents a fraction of stable OC, while TOC represents the total belowground OC. The variable amounts of other fractions of OC explains the observed difference in trends (Lebron et al., 2024). Inorganic carbon from LOI is higher than TIC% from EA, which could be explained due to the potential for loss of additional minerals alongside CaCO_3_, causing overestimation of CaCO_3_. The opposite trends observed with increasing distance from marsh margin in SOC and TOC reveal the importance of detailing OC laboratory methods on determining within-site OC distribution patterns (Fig. 5a, e.g. with increasing distance from marsh margin). Specifically, it is recommended that methodologies for OC stocks assessments describe the presence or removal of roots to account for belowground OC or soil OC measurements.

The site-wide CD mean in this study at the marsh site within the Rogerstown estuary is 11.1 ± 4.2 kg m^−3^ at 10-cm depth. To compare our methodology against existing estimates, this value is less than half that of Penk (2019) who estimated a minimum belowground soil CD of 25.9 C kg m^3^ for Irish salt marshes using LOI at 550 °C and the Craft (1991) conversion. Likewise, the regional OC stock estimate for Irish salt marshes in this study is 76,923 t C to 10-cm depth, based on the area stated in Penk (2019) of 69.3 km^2^, which is around 23–43% of Penks’ estimate of 179,000–340,000 t C. These disparities primarily arise from the methodology and potentially some magnification of error in upscaling (Ladd et al., 2022). For instance, CD here is based on a stable fraction of SOC, whereas Penk’s method can cause some overestimation and uncertainty (Lebron et al., 2024; Smeaton et al., 2022a). Such overestimation when upscaled, combined with the potentially smaller fraction of SOC represented in this study, may explain this gap. Thus, it is important to consider the methodology used, highlighting the OC pool being measured and the temperature of LOI for clarity. It is also important to consider and further investigate potential errors from over- or underestimation that may be magnified in upscaling. Like this study, Penk’s (2019) estimates considered east coast marshes, which are mostly estuarine and mud/sand sediment (Curtis & Skeffington, 1998). The more exposed, high-energy west coast marshes and the predominantly sandy north-western marshes require site-specific estimates. Penk (2019) highlighted that surficial estimates at 10 cm depth are low due to the varying soil depths of Irish salt marshes.

At marsh-scale in Ireland, the CD value using our modified LOI method compares to Burke et al., (2022; using Craft, 1991) who found, ~ 11 kg m^−3^ scaled to 10 cm (underestimated when scaled, due to lower CD at depth), placing our estimate in the existing range for Dublin marshes. They also noted that Rogerstown estuary CD is higher, thus highlighting methodological differences between their LOI 550 °C and Craft (1991) conversion and our method as described above. The standard deviation (Stdev) in this study falls within the low marsh belowground SOC content range in the UK as per Smeaton et al., (2022b; Stdev ~ 0.1–5.1; Supplementary material Table 1).

To provide a comparison against other Irish and UK studies, as well as to facilitate further investigation of marsh-wide CD estimation, this study offers a conversion factor for Irish marshes between TOC% from EA and OM% from LOI (Fig. 4; following Austin et al., 2022; Smeaton et al., 2022a). This study found TOC (%) = 0.398 × OM (%) − 0.474. The correlation between TOC% (EA) and OM (LOI) was *R*^2^ = 0.74, *p* < 0.01; Fig. 4). This is just lower than the correlations calculated for UK marshes, where *R*^2^ = 0.78–0.83. (Austin et al., 2022; Smeaton et al., 2022a). The *y*-intercept in this study was lower than those of the UK marshes (OC% = 1.45–2.08), and slope was just higher (slope = 0.37–0.38). As a further brief demonstration of methodological differences in OC stock estimates, data from our study was used to compare common SOC methods (Supplementary Table 2). The mean CD estimate from our TOC% conversion factor (EA) is 25.1 kg m^−3^ (regional salt marsh stock = 173,943 t C based on Penk’s (2019) Irish salt marsh area) at 10-cm depth. This estimate is around 51 to 97% of Penk’s range. Using the Craft (1991) conversion from this study’s TOM results produces the highest mean CD at 30.1 kg m^−3^ (regional stock = 208,593 t C) which is 61 to 116% of Penk’s range. This highlights that particular care is required when comparing OC estimates from different methodologies.

Future research is required to understand the interactions and relative influence of the potential controlling factors in this study on the spatial distribution of within-site SOC and CD. Specifically, the degree to which within-site SOC and CD variation is influenced by environmental and geomorphological factors and which, if any, factors have the most important influence on controlling the distribution of carbon storage at local scales.

### The impact of sampling strategy on marsh-wide belowground organic carbon density estimates

Sample sizes for carbon storage studies are often limited and sample locations spread across large areas (e.g. global estimates using 24–99 studies, national studies using 15–26 marshes or studies taking an average of two samples per marsh; Table 1). This study demonstrates that sampling strategy and size impact the likelihood of obtaining a marsh-wide CD which was not statistically different (H_0_ for two-sample *t* test accepted, 95% confidence) from various subsamples and sampling strategies (*n* = 5, 10, 15 and 20) to the site-wide CD in this study (established using all *n* = 60 samples). Random sampling is recommended as the best strategy for various sample sizes (*n* = 5, 10, 15 and 20) to reduce the risk of a biased dataset (difference in means between subsamples and the full site-wide CD in this study was < 0.3 kg m^−3^ for all datasets, Fig. 8a).

We found that restricting datasets to the marsh margin or within 20 m of the creek may cause bias in the dataset (e.g. 10 to 80% of subsets statistically different from the measured mean for *n* = 5, Table 3). In this study, sampling near the marsh margin overestimated marsh-wide CD by a factor of 0.55, where CD = 16.2 kg m^−3^ (Fig. 8a). Conversely, avoiding marsh boundaries caused an underestimation in mean CD by a factor of 0.1 (Fig. 7a). When upscaled to regional or national estimates, this could have a significant impact on the total OC stock estimates as demonstrated by Austin et al. (2022). At the marsh edge, higher SOC is found in this study, which was also seen in previous studies at regional scales, for example in a Scottish salt marsh (Miller et al., 2023; Fig. 4a). As discussed above, such overestimation of CD at the marsh margin could be due to various factors that may contribute to high CD at the marsh margin, such as tidal inundation, vegetation species distribution and sediment deposition (French et al., 1995; Moeller et al*.,* 1999, 2001; Penk & Perrin, 2022).

Smeaton et al. (2022a) highlights the importance of understanding the OC estimate ranges in European marshes, since carbon accumulation rates have also traditionally been overestimated when included in global inventories. While the OC accumulation rates may be lower than previously thought, this study and recent stock estimates highlight the value of the OC already buried in the marsh. The local variation in OC distribution (SOC, CD) is important; however, the carbon stock estimates in Irish studies can vary by up to 43% depending on the method chosen for measurement and when upscaled (Fig. 6a; Penk & Perrin, 2022; Smeaton et al., 2022a). Thus, sampling strategy and methodology is important to reduce and quantify the uncertainty from biased sampling strategies that over- or underestimate SOC stocks and CD values and improve regional and national scale OC stock assessments (Austin et al., 2021; Smeaton et al., 2022a, 2023; 2024). Furthermore, Ladd et al. (2022) calculated up-scaled SOC estimates and found that sampling depth and upscaling technique led to up to 52 times variation in marsh-wide SOC stocks. The largest difference arose from SOC stocks measured to 1-m depth (recommended standard, Howard, 2014), compared with the determined marsh sediment depth (minus mudflat sediment). Overestimation occurred due to the elevated TOM content of mudflat sediments. Combined influence from sampling depth, upscaling technique, sampling strategy and sample sizes is thus important. The recommendations herein advise a best practice to capture marsh-wide SOC and CD, utilising random sampling and appropriate sample sizes to augment the current best practices for the upscaling of within-site SOC stocks and CD estimates to regional and national scales.

Carbon credit initiatives such as the recently developed salt marsh carbon code in the UK will enable businesses to voluntarily purchase salt marsh carbon, using the carbon market to offset CO_2_ emissions and fund salt marsh restoration projects (Trouwloon et al., 2023; UKCEH, n.d.). An example of the use of carbon credits is carbon neutrality achievement claims, which clearly state the past goals accomplished, and rely on carbon offsetting (using credits for compensation). The claims can be used as a strategy to mitigate climate change impacts, by aiming to achieve “no net increase” in emissions (Trouwloon et al., 2023). The recommendations here are imperative to enable carbon credits to be used effectively for such compensation. Beaumont et al. (2014) highlighted the uncertainty involved in valuing salt marsh carbon; thus, quantifying the uncertainty in national and regional OC stock assessments (SOC, CD, TOC) is vital for carbon credits schemes. This study found that the largest error for the subsample CD site estimate for small sample sizes (*n* < 20 per ~ 800 m^2^ site) compared to the site-wide CD was an overestimation by a factor of 1.75 (mean CD = 19.4 kg m^−3^; Fig. 7a). Such an overestimation would impact the assessment of carbon credits required for offsetting CO_2_ emissions; thus, the quantifications in this study can be used to an inform sampling strategy planning for CD measurements. To further decrease the uncertainty in carbon storage estimates, future work is required to understand the key drivers of SOC/TOC storage (sedimentary, hydrodynamics, biomass (AGB, BGB)) and to quantify whether processes have a dominant influence on the distribution of SOC at local scales (10 s–100 s m).

## Conclusion

The site-wide CD mean estimated in this study at the salt marsh site in the Rogerstown estuary, a small (~ 800 m^2^) salt marsh on the east coast of Ireland, is 11.1 ± 4.2 kg m^−3^ at 10-cm depth, ranging from 5.2 to 22 kg m^−3^. The standard deviation is comparable to SOC for low marsh sites in Great Britain. This value is lower than existing estimates for Ireland, based on west coast marshes due to our methodology, highlighting the importance of careful definition of what is measured in each case. There is also potential for overestimation in methodologies using higher LOI temperatures for TOM and the SOC conversion using Craft (1991), compared with the LOI calculation utilised in this study, which measures a stable OC fraction of the TOM; thus, it is important to consider methodologies when calculating SOC stocks. Our method provides a site-specific alternative and comparison to common methodologies, which have been noted to cause overestimation, and in case EA is not feasible due to availability or expense.

CD varies within the salt marsh site by up to 423%, peaking along the marsh edge where it exceeded the marsh interior by 174%. The largest error for the ‘subsample CD’ site estimate when sampled within 5 m of the marsh margin (*n* < 20 per ~ 800 m^2^ site) was an overestimation by a factor of 1.75, compared to the site-wide CD estimate. These results demonstrate the dominant influence of processes at the marsh edge such as sediment supply and deposition, tidal inundation and vegetation composition. Additionally, this study quantifies the impact of sampling methods on the site-wide CD estimate at a local scale. It is recommended that within-site measurements of small sample sizes (*n* < 20) should be randomly sampled and not clustered around a marsh margin to obtain the full variation of marsh-wide SOC (as measured in this study using *n* = 60 samples). Furthermore, these results can be utilised to improve the efficiency of sampling campaigns. They demonstrate that smaller datasets (*n* < 20) can achieve a mean CD which is not statistically different (*t* test *H*_0_ accepted at 95% confidence) when compared to the mean determined from a larger sample grid (*n* = 60), if sampled randomly without clustering and without excluding marsh and the creek edges. These findings provide a best practice to capture marsh-wide SOC and CD, utilising random sampling and appropriate sample sizes to augment the current best practices for the upscaling of within-site SOC stocks and CD estimates to regional and national scales.

These results provide an empirical basis for planning a sampling campaign for salt marsh carbon storage, demonstrating the impact of sample location within a salt marsh site and the impact of scaling up from local-scale measurements. Secondly, salt marsh carbon storage plays a major role in the mitigation of global climate change impacts, and the quantification of the uncertainty in carbon estimates (SOC stocks and CD) is therefore vital to constraining uncertainties in scaled-up national and regional OC stock inventories. Future work is required to investigate the interactions of key drivers of the distribution SOC burial in salt marshes at local scales (10 s–100 s m). Such investigation should include whether and to what extent the within-site variation can be explained by factors that are already known to be involved in carbon burial (e.g. biomass (AGB, BGB), elevation/hydroperiod, soil moisture).

## Supplementary Information

Below is the link to the electronic supplementary material.

ESM1 (DOCX 4.38 MB)

## Data Availability

The datasets generated during and/or analysed during the current study are not publicly available at present due to being part of an ongoing PhD project, but are available from the corresponding author on reasonable request. Supplementary materials for some datasets have been provided.
