# Supplementary material for: Spatial distribution of soil organic carbon in an Irish salt marsh (Rogerstown Estuary)
Source: Environ Monit Assess. 2025 Jul 10;197(8):899. doi: 10.1007/s10661-025-14250-9 (PMC12245998; doi:10.1007/s10661-025-14250-9)
Supplement: Supplementary file 1 — (DOCX 4.38 MB) [file 10661_2025_14250_MOESM1_ESM.docx]

**Spatial distribution of soil organic carbon in an Irish salt marsh (Rogerstown Estuary)**

Juliet Rounce^1*^, Iris Möller^1^ and Andrew J Manning^2,3^

^1^ Department of Geography, Trinity College Dublin, Dublin, Ireland, ^2^HR Wallingford Ltd, Estuaries & Dredging group, Howbery Park, Wallingford, Oxon, UK. OX10 8BA Centre for Coastal Dynamics and Engineering (C-CoDE), ^3^Coastal Processes Research Group, School of Earth, Ocean & Env. Sciences, University of Plymouth, UK

*corresponding author: [rouncej@tcd.ie](mailto:rouncej@tcd.ie)

**Supplementary Materials**

**Table 1** The range of belowground soil organic carbon (OC) content and standard deviation in narrow core samples at 10-12 cm depth on natural marshes around the UK. Summarised data from Smeaton *et al*. (2022b).


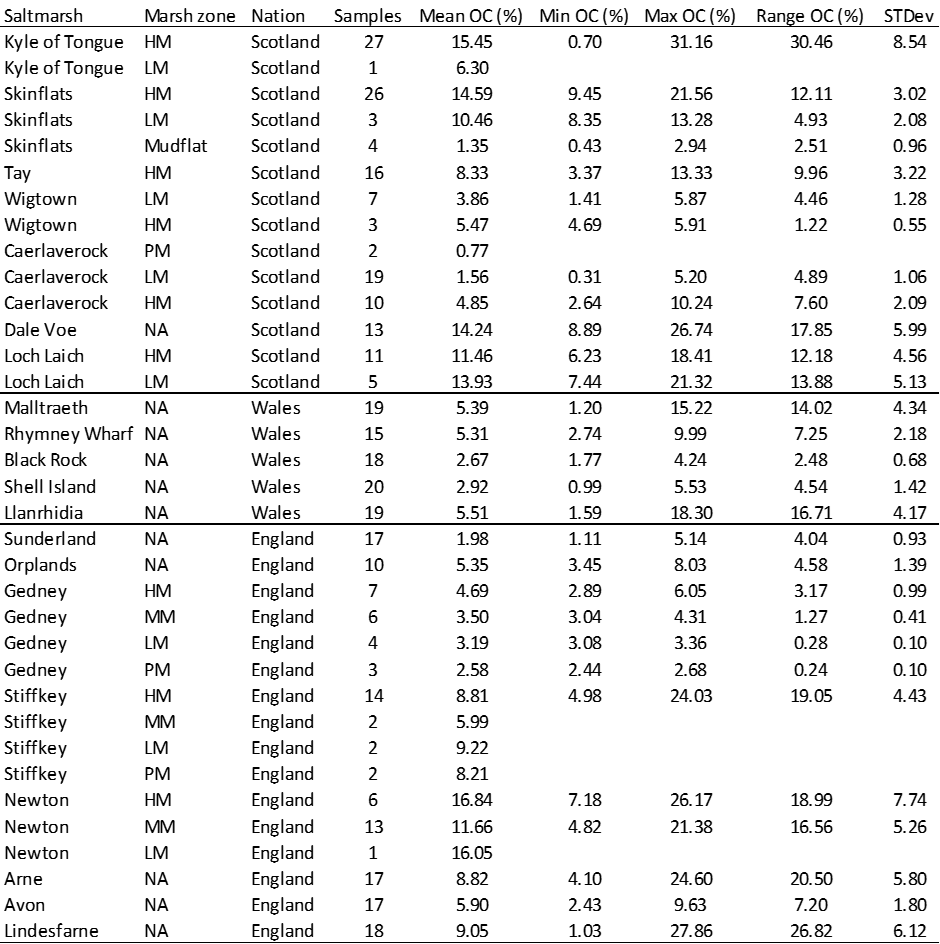


**2. Methods**

**Sequential loss on ignition** (Modified Cambridge Protocol; University of Cambridge, 2022).

Loss-on-ignition protocol: Subsamples were taken using a 2 cm^3^ volumetric sampler. The subsamples were heated in an oven at 65°C for >24 hours to remove moisture, after obtaining the wet sample mass. The samples were then weighed using the same scales to obtain moisture content (MC). After weighing, the samples heated in a furnace at 400°C for 6 hours. The samples were weighed the following day and returned to the furnace at 480°C for 6 hours to obtain weights for organic matter content (OM) and carbon content (SOC). This was repeated the following day at 950°C for 6 hours to obtain the final weight for carbonate content (CaCO_3_). These parameters were calculated as follows:

***MC***

Water logging increases decomposition rate, therefore, moisture content (MC) will be assessed as an indication of retention potential of carbon (e.g. Hemminga et al., 1991). The MC (Eqn 1), representing pore water content, may also be utilised to calculate additional parameters such as porosity and dry bulk density.

*MC (%) = (FS – OD)/FS*100* **Equation 1**

Where *FS* = fresh sample (g), before drying, *OD* = oven dried at 65°C (g), after heating.

***OM***

Organic matter (OM), representing mass lost post-heating as a percentage of initial dry weight (Eqn 2; e.g. Davies 1974; Ball 1964), may be utilised to calculate SOC content.

*OM (%) = OD – AR480/OD*100* **Equation 2**

Where ARX = ash residue at X°C.

***CaCO_3_***

CaCO_3_, as a percentage of initial dry weight, utilises a conversion factor using a ratio of the molecular weights of CaCO_3_ to CO_2_ lost (Eqn 3, University of Cambridge, 2022):

CaCO_3_ (%) = ((AR480-AR950) * 2.274) / OD*100 **Equation 3**

***Carbon content (coal)***

The SOC, represented as a percentage of initial dry weight, utilises the mass lost between ash residue at 400°C and 480°C to distinguish between total OM and the carbon fraction (Eqn 4).

Coal (%) = (AR400-AR480)/ OD*100  **Equation 4**

**Bulk Density**


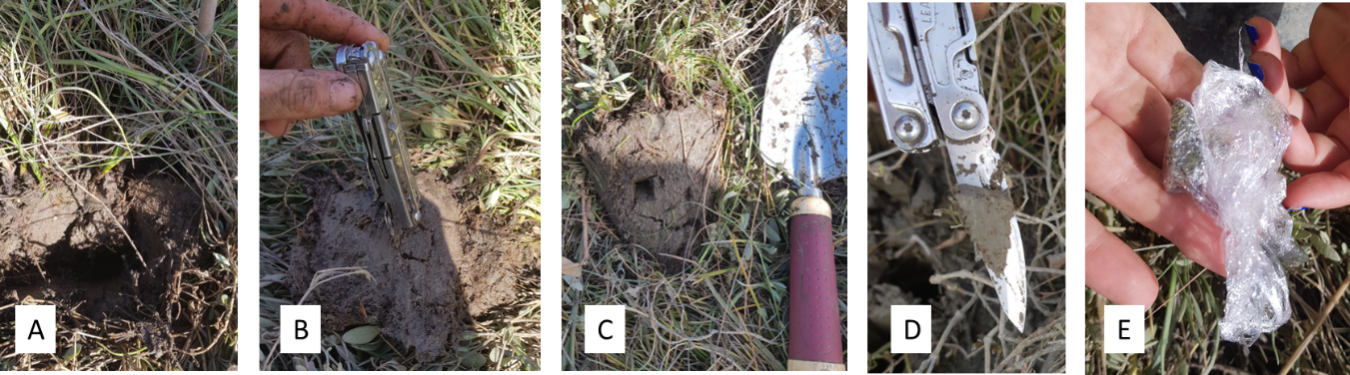


**Supplementary Material Fig 1** Field and lab methods for bulk density A-E - careful field sampling with a penknife from a subsample. F-I – laboratory analysis for bulk density, volume.


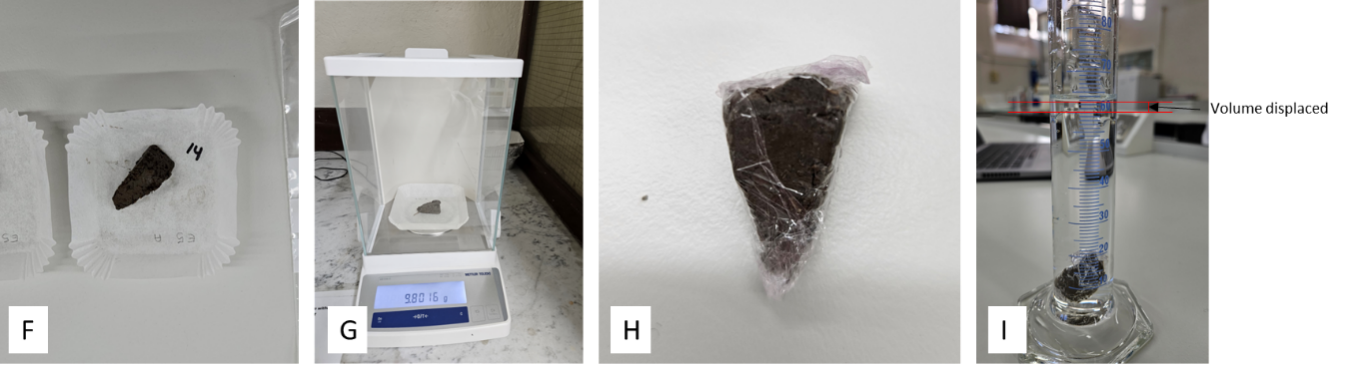


1. **Methods *Particle Size Analysis***

Subsamples from the sediment cores were prepped for PSA by removing organics and carbonates (August-September 2024; Bartminski *et al*., 2022; Sperazza *et al*. (2004), Eshel *et al*., 2004 and Gray *et al*., 2010). The sample (~0.5 g) was covered in de-ionised water to disperse the particles, then H_2_0­_2_ 30% solution was added for reduction. In the water bath, the samples were heated at 50 ˚C for 1 hour, 60 ˚C for 1 hour, 70 ˚C for 2 hours and 80 ˚C for 1 hour until the reaction was complete. The samples were centrifuged at 4000 rpm for 5 minutes with H_2_0_2_ and rinsed with 40 ml de-ionised water, repeated twice. 20 ml HCl 10% was added to remove the carbonates, then rinsed and centrifuged at 4000 rpm with 20 ml de-ionised water three times. Finally, the samples were centrifuged and dried in the oven at 50 ˚C for 48 hrs. De-flocculant 20ml 50g/l Sodium hexametaphosphate solution added overnight. Samples agitated on roller mixers for 90 min, then sieved (1 mm) prior to PSA. Samples were analysed in the Mastersizer 3000 (obscuration 5%, refractive index 1.520, particle density 1.05 g cm^-^³).

**4. Discussion *Methods comparison for SOC***

**Table 2** Comparison of soil organic carbon estimates at 10 cm depth using the data from this study for 3 methods. 1. The LOI method in this study for direct SOC estimate, 2. The conversion factor from this study of OM % to TOC % from elemental analysis and 3. the conversion of this study’s OM % to SOC % using the Craft (1991) conversion (SOC % = 0.40 * LOI % + 0.0025 * (LOI %^2^)).

**References**

Ball, D. F. (1964). Loss-on-Ignition as an Estimate of Organic Matter and Organic Carbon in Non-Calcareous Soils. Journal of Soil Science, 15(1), 84–92. https://doi.org/10.1111/j.1365-2389.1964.tb00247.x

Bartmiński, P., Świtoniak, M., Drewnik, M., Kowalska, J. B., Sowiński, P., Żyła, M., & Bieganowski, A. (2022). Methodological problems with the classifi cation and measurement of soils containing carbonates. | EBSCOhost. Soil Science Annual, 73(1), 1–10. https://doi.org/10.37501/soilsa/149235

BPI Consulting LLC. (2024). Anderson-Darling Test for normality June 2011 [Excel workbook download]. https://www.spcforexcel.com/knowledge/basic-statistics/anderson-darling-test-for-normality (Accessed: 18/07/2024)

Davies, B. E. (1974). Loss-on-Ignition as an Estimate of Soil Organic Matter. Soil Science Society of America Journal, 38(1), 150–151. https://doi.org/10.2136/sssaj1974.03615995003800010046x

Eshel, G., Levy, G. J., Mingelgrin, U., & Singer, M. J. (2004). Critical Evaluation of the Use of Laser Diffraction for Particle-Size Distribution Analysis. Soil Science Society of America Journal, 68(3), 736–743. https://doi.org/10.2136/sssaj2004.7360

Gray, A. B., Pasternack, G. B., Watson, E. B., Gray, A. B., Pasternack, G. B., & Watson, E. B. (2010). Hydrogen peroxide treatment effects on the particle size distribution of alluvial and marsh sediments. The Holocene, 20(2), Article 2. https://doi.org/10.1177/0959683609350390

Hemminga, M. A., de Leeuw, J., de Munck, W., & Koutstaal, B. P. (1991). Decomposition in Estuarine Salt Marshes: The Effect of Soil Salinity and Soil Water Content. Vegetatio, 94(1), 25–33. https://www.jstor.org/stable/20038787

Smeaton, C., Ladd, C. J. T., Havelock, G. M., Miller, L. C., Garrett, E., Hiles, W., McMahon, L., Mills, R. T. E., Radbourne, A., Rees-Hughes, L., Riegel, S., Barlow, N. L. M., Skov, M. W., Gehrels, R., & Austin, W. E. N. (2022b). Physical and geochemical properties of saltmarsh soils from narrow diameter gouge cores in UK saltmarshes collected between 2018 and 2021 [Text/csv Comma-separated values (CSV)]. NERC EDS Environmental Information Data Centre. https://doi.org/10.5285/D301C5F5-77F5-41BA-934E-A80E1293D4CD (Accessed: 17/01/2024)

Sperazza, M., Moore, J. N., & Hendrix, M. S. (2004). High-Resolution Particle Size Analysis of Naturally Occurring Very Fine-Grained Sediment Through Laser Diffractometry. Journal of Sedimentary Research, 74(5), 736–743. https://doi.org/10.1306/031104740736

University of Cambridge. (2022). Department of Geography Loss on Ignition. https://www.geog.cam.ac.uk/facilities/laboratories/techniques/loi.html (Accessed: 23/08/2022)
